# The Complex Architecture of Plant Cuticles and Its Relation to Multiple Biological Functions

**DOI:** 10.3389/fpls.2021.782773

**Published:** 2021-12-10

**Authors:** Nicolas Reynoud, Johann Petit, Cécile Bres, Marc Lahaye, Christophe Rothan, Didier Marion, Bénédicte Bakan

**Affiliations:** ^1^INRAE, Unité Biopolymères, Interactions, Assemblages, Nantes, France; ^2^INRAE, University of Bordeaux, UMR BFP, Villenave d’Ornon, France

**Keywords:** plant cuticle, architecture–function relationship, cutin, cell wall polysaccharides, phenolics

## Abstract

Terrestrialization of vascular plants, i.e., Angiosperm, is associated with the development of cuticular barriers that prevent biotic and abiotic stresses and support plant growth and development. To fulfill these multiple functions, cuticles have developed a unique supramolecular and dynamic assembly of molecules and macromolecules. Plant cuticles are not only an assembly of lipid compounds, i.e., waxes and cutin polyester, as generally presented in the literature, but also of polysaccharides and phenolic compounds, each fulfilling a role dependent on the presence of the others. This mini-review is focused on recent developments and hypotheses on cuticle architecture–function relationships through the prism of non-lipid components, i.e., cuticle-embedded polysaccharides and polyester-bound phenolics.

## Introduction

Terrestrial colonization of plants came along with the development of four sophisticated hydrophobic macromolecular assemblies, i.e., cuticle, suberin, lignin and sporopollenin (Yeats and Rose, 2013; Graça, 2015; Li et al., 2019; Ralph et al., 2019), which enabled plants to resist to the harsh conditions of the environment, to stiffen their architecture, to ensure nutrient and water adsorption, their reproduction and land dispersion. Cuticle has a high plasticity, especially adapted to organ growth. This plasticity was recently illustrated in the case of the cuticle of lateral root primordia which controls further lateral root emergence (Berhin et al., 2019). Indeed, any defects in the cuticle biosynthetic pathways induce defects in cuticle assembly and impact organ growth and morphology (Fich et al., 2016; Berhin et al., 2019). Cuticle fulfills multiple functions, e.g., in the control of water and gas exchanges, in the defense signaling against biotic and abiotic stresses, in plant development with many interactions with hormone signaling and cell wall biosynthesis, in the protection against UV radiation, in the retention of environmental pollutants, in the induction of responses to mechanical stimuli, and constitute an habitat for the plant microbiome (Schönherr and Riederer, 1989; Martin and Rose, 2014; Meder et al., 2018; Cordovez et al., 2019). These multiple functions impact crop yields and quality, including post-harvest quality and processing which has boosted research on the structure, assembly, and biosynthesis of their macromolecular and molecular constituents. Finally, recent data showed that cuticle functions could not be regarded only through the lens of their chemical composition, but resulted from a spatial organization of molecules and macromolecules, i.e., a 3D architecture, finely regulated at the genetic and physiological levels. This mini-review reports recent data on cuticle structural features and the ensuing hypotheses on its architecture, its evolution during organ development and its relationship with their functional properties.

## From Molecular Diversity to Cuticle Architecture

Plant cuticles are composed of three main types of chemical components, i.e., lipids, carbohydrates, and phenolics. Lipids give the cuticles their hydrophobic properties. They consist of molecules easily extractable with organic solvents, i.e., epi- and intra-cuticular waxes, dispersed at the surface and within the cuticles, respectively, and of insoluble lipid polymers, i.e., cutan and cutin. While cutan polymer contains non-hydrolysable bounds that limit structural investigation (Bhanot et al., 2021), cutin is a polyester of oxygenated fatty acids (mainly with hydroxyl and/or epoxide groups, but in less proportion with oxo groups) of 16 and 18 carbon atoms (Yeats and Rose, 2013). In some plants and, especially those of the Brassicaceae family, high amounts of dicarboxylic acids (DCA) are found while they are minor compounds in other plants (Franke et al., 2005; Razeq et al., 2021).

The diversity of cutin monomer compositions has to be analyzed in relation to the architecture of the cutin polyester. Two physical parameters characterize the architecture of polymers, their size and their reticulation (Caccavo et al., 2018). In contrast with synthetic polyesters, measuring precisely the size of the cutin polymer is not possible since it cannot be solubilized. However, *in planta*, the cutin synthase (CUS1)-catalyzed cutin polymerization from 2-monoacylglycerol (2-MAG), gives rise to polyester with glycerol at the carboxylate terminus (Yeats and Rose, 2013). Therefore, the molar ratio of glycerol to hydroxy-fatty acid (HOFA) ratio allows the comparison of the polymer sizes of cutins between plant species or mutants. Indeed, this ratio is highly variable between plants suggesting a diversity of cutin molecular sizes (Graça et al., 2002) and in *cus1* tomato mutants, the increase of this ratio regarding the wild-type suggested a lower cutin polymer size (Philippe et al., 2016). This chemical rule has however some limit for DCA-rich cutins of Brassicaceae, where this particular composition came along with a high amount of glycerol and a DCA:glycerol molar ratio of 1:2 consistent with the formation of glycerol-DCA-glycerol polyesters (Yang et al., 2016).

Concerning cutin reticulation, it is necessary to consider the basic chemical reaction of polyesterification of components bearing both hydroxyl and carboxylate groups and two carboxylate groups and leading to branched polyesters (McKee et al., 2005) or cross-linked polymer networks (Gu et al., 2019). Branched vs. linear cutin polyesters, will depend (i) on the presence of mid-chain hydroxyls as for the 9(10),16-dihydroxy hexadecanoic acid (diOHC16) and (ii) on the OH/COOH molar ratio in the global monomer composition and especially on the level of DCA. It is also important to consider the hydroxyls of glycerol in this ratio, although this triol displays relatively low contents in cuticles. Indeed, as a branching point, it seems involved in the reticulation of tomato cutin (Philippe et al., 2016).

**FIGURE 1:**
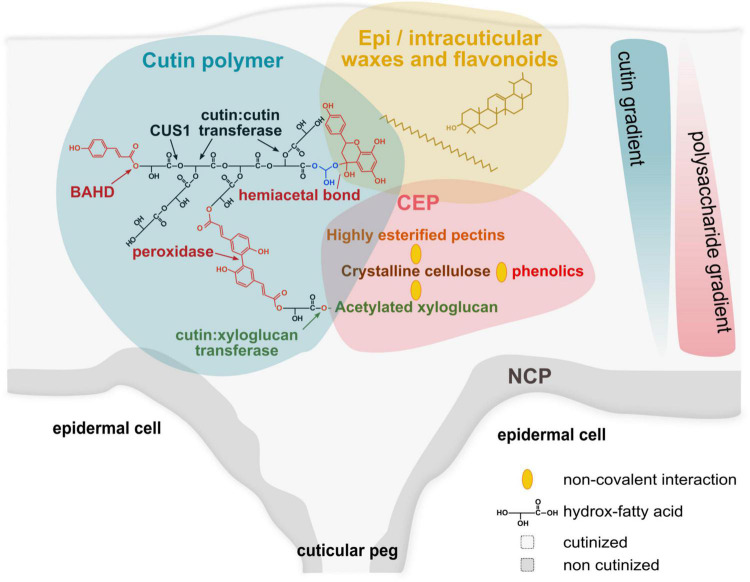
Schematic model for the complex architecture of plant cuticles. Plant cuticle (gray background) is a natural hydrophobic composite. The lipid scaffold of the cuticle is the cutin polymer (blue background) embedded with waxes (yellow background), and associated to the cuticle-embedded polysaccharides (CEP, red background). Cutin is a polyester network of HOFA mainly formed by the activities of CUS1 (a protein from the multigenic GDSL family of esterase/lipase) (Girard et al., 2012) and cutin:cutin acyl transferase (CCAT, the protein associated with this activity has not yet been identified) during the expansion of the plant cuticle (Xin et al., 2021). Para-coumaric acid esterification of the primary alcohol group of HOFA (co-position) is catalyzed by an enzyme of the multigenic BAHD family (Molina and Kosma, 2015) and could form covalent bridges between the polyester chains through peroxidase oxidation (Kerr and Fry, 2004; Arrieta-Baez and Stark, 2006). The strong association of flavonoids (e.g., naringenin and naringenin chalcone in tomato), especially at the end of fruit growth (Hunt and Baker, 1980) could involve hemiacetal bond with primary hydroxyl groups (e.g., with the glycerol end residue of 2-monoacylglycerol, the precursor of CUS1). The cutin:xyloglucan transferase is a good candidate to link covalently the cutin network to the CEP (Xin and Fry, 2021). In contrast to the non-cutinized polysaccharides (NCP), the CEP concentrates crystalline cellulose, highly esterified pectins and acetylated xyloglucans (Philippe et al., 2020) associated by non-covalent bonds (yellow ellipses). In addition, the hydrophobicity of these polysaccharides is compatible with non-covalent interactions (Ngouémazong et al., 2015; Dai et al., 2020; Lindman et al., 2021) with the cutin network and intracuticular waxes. Finally, cellulose can also interact with phenolics (Phan et al., 2015). The cutin network and CEP form gradients in the cuticle thickness create a spatial heterogeneity in the plant cuticles.

However, while tomato cutin contains more than 75% diOHC16, it is surprising to observe that more than 80% of the midOH groups of tomato cutin are esterified and can reach up to 90% in the cutin of red ripe fruit (Philippe et al., 2016). Furthermore, in the *cus1* mutant, the lower expression of CUS1 induces a high decrease of midOH esterification (Philippe et al., 2016). In these mutants, the cutin deposition and cutin density are also lowered suggesting that CUS1 could also facilitate esterification of the mid-chain secondary alcohol group when the cutin density is sufficient to create a favorable hydrophobic environment (Girard et al., 2012; Philippe et al., 2016). Esterification of midchain hydroxyl could also involve another enzyme and especially a cutin: cutin transacylase activity recently described (Xin et al., 2021). Whatever the mechanism of polymerization, in diOHC16-rich cutins, the polyesterification would typically lead to hyperbranched macromolecules that are easily soluble in organic solvents (Testud et al., 2017) while cutin is insoluble. Furthermore, branching through the midOH group should lead to an increase of non-esterified ω-OH groups while this was not observed (Philippe et al., 2016). It is therefore necessary to consider the links between cutin and the other cuticle compounds, i.e., phenolics and polysaccharides (Figure 1).

Phenolics, generally regarded as minor components, are also embedded in the cuticles. These phenolics comprise two main types of molecules, phenolic acids (e.g., para-coumarate) and flavonoids (e.g., naringenin). Hunt and Baker (1980) suggested that they are associated with the cutin by ester bonds since all the phenolic acids are released after alkaline hydrolysis (Hunt and Baker, 1980). However, ester links with polysaccharides is also possible and are described in the xylan and pectin fractions of the cell walls of both monocots and dicots (Mnich et al., 2020). An ester link with the cutin polymer must be also considered regarding the characterization of an enzyme of the BAHD family capable to esterify the ω-position of HOFA by a phenolic acid (Rautengarten et al., 2012; Molina and Kosma, 2015). Unexpectedly, these minor ester bonds of the cutin polymer seem essential for the cuticle architecture of tomato fruit (Lashbrooke et al., 2016). The importance of such chemical bonds is also strengthened by the evolution of plant hydrophobic polymers, and especially in mosses where cutin is a co-polyester of HOFA and phenolic acids (Renault et al., 2017). On the contrary, a part of flavonoids is extractible with methanol while the other was extracted only after alkaline hydrolysis of the cuticle (Hunt and Baker, 1980). Ester bonds between cutin and flavonoids are unlikely due to the absence of carboxylate on these molecules. From sorption studies of naringenin into tomato cuticles, it was suggested that flavonoids can form solid clusters within the cuticles which can be dissociated only after cutin depolymerization (Dominguez et al., 2009). In agreement with this equilibrium partition within the cuticle, the ketone group of flavonoids could form hemiacetal and/or acetal derivatives with the alcohol group of cutin, and especially with the glycerol moieties of cutins (Moity et al., 2015) (Figure 1). In this regard, such bonds can be also considered with minor cutin fatty acids containing oxo groups.

The ester bonds between phenolic acids and cutin could be questioned regarding cutin reticulation. These HOFA-phenolic esters could dimerize through carbon–carbon bonds under oxidative reactions as suggested for suberin (Arrieta-Baez and Stark, 2006). As for arabinoxylan cross-linking (Kerr and Fry, 2004), a similar chemical mechanism could be considered for the anchoring of polysaccharides and cutin (though phenolic acids in plant cell walls are commonly found on arabinose moieties that are not a dominant sugar in tomato cuticle) and/or for the cross-linking of cutin linear chains. The role of phenolics is strengthened by the polyesterification of diOHC16 and phenolic-rich fractions of tomato pomaces. This non-catalyzed and temperature-controlled process induces oxidation of the phenolic compounds and the formation of an insoluble cross-linked polymer network and not of soluble branched polyesters (Marc et al., 2021). *In planta*, this oxidation could be driven by peroxidases that accumulate at the end of fruit growth (Andrews et al., 2000).

The presence of polysaccharides in the cuticular layer leads to the concept of the cell wall continuum of the epidermis (Fich et al., 2016). The fine structure of the cutin-embedded polysaccharides (CEP) was first investigated by immunolabeling studies on leaves or fruit epidermis from pear and tomato and resulted in either very faint or no labeling of cellulose and pectin within the cutin matrix (Guzman et al., 2014a,b; Segado et al., 2016) probably due to the masking of the glycoside epitopes. By combining different investigation methods, it was recently shown that these CEP display specific features regarding those of the sub-cuticular, non-cutinized cell walls. Especially, in the tomato cuticle, the pectin and hemicelluloses (enriched in xyloglucan) are highly esterified, while a higher content in crystalline cellulose is observed (Philippe et al., 2020). Pectins embedded in the cuticle display a low ramification of rhamnogalacturonan (RGI). These CEP structural features are in favor of a lipid-polysaccharide association. Indeed, high methyl- and acetyl-esterification of pectin and hemicellulose increase their apolarity making interactions with lipids possible. Indeed, pectin methyl- and acetyl-esters have often been related to oil/water emulsification and emulsion stabilization (Ngouémazong et al., 2015). Low ramified RGI in the CEP with the apolar rhamnose residue and the acetyl esters are also expected to participate in pectin–lipid interactions. Cellulose is an amphiphilic polysaccharide that stabilizes lipid emulsion through hydrophobic interactions (Lindman et al., 2021). Its crystalline assemblies also interact with lipids to stabilize emulsions via the hydrophobic face of the crystals (Dai et al., 2020). Furthermore, phenolic derivatives of lipids can also contribute to lipid–polysaccharides interactions since phenolics can interact with cellulose (Phan et al., 2015). Thus, lipids and derivatives can physically form hydrophobic association with polysaccharides in the cutinized cell-wall domain of the cuticle. As observed for the xyloglucan-cellulose-pectin assemblies of cell walls, the non-covalent links between the polysaccharides and cutin should be sufficient to give the cuticle both strength and flexibility needed for organ growth.

If a covalent link was highlighted after partial depolymerization of lime fruit (Tian et al., 2008), no other studies reported similar heteromers in plant cuticles. Interestingly, an enzyme activity capable of linking HOFA and polysaccharides, and specific for xyloglucans, i.e., a cutin:xyloglucan transacylase (CXT), was recently characterized (Xin and Fry, 2021). Furthermore, in tomato fruit, both the CXT activity and xyloglucan contents increase in the epidermis during fruit expansion (Takizawa et al., 2014). CXT could play a significant role in the construction of the cuticle architecture since xyloglucans are embedded in cuticles (Philippe et al., 2020).

In summary, the cutin polyester can be considered as a branched or hyperbranched polyester architecture which is insolubilized by the multiple covalent and non-covalent links created during organ growth with polysaccharides and phenolics (Figure 1). The cuticle image portrayed from numerous microscopy investigations described cuticle architecture as two hierarchically organized layers including an upper cuticular proper mixing cutin and waxes, and a lower cuticular layer containing cutin, polysaccharides, and intra-cuticular waxes (Jeffree, 2006). Much progress has been done in imaging, especially thanks to the development of Raman, infrared and MALDI mass imaging that provide information on the distribution of lipid, carbohydrate and phenolic compounds within cuticles (Vrkoslav et al., 2010; Velickovic et al., 2014; Philippe et al., 2016; Sasani et al., 2021). Actually, compositional gradients were observed both on the surface (Philippe et al., 2016) and within the thickness of the cuticular layer (Philippe et al., 2020; Sasani et al., 2021). The cutin/polysaccharide ratio decreases from the outer cuticle to the inner cell wall-cuticle interface (Philippe et al., 2020) while phenolic compounds are mainly associated with the waxes and cutin-rich regions (Sasani et al., 2021). The question of the lipid-polysaccharides molecular orientational order was also raised recently on leaves models (Hama et al., 2019). Furthermore, deep cutinization of cell walls can occur between epidermal cells creating anticlinal pegs adjacent to epidermis cells (Buda et al., 2009). Discontinuities can also occur with the presence of trichomes creating small polysaccharide transcuticular channels (Fich et al., 2020) or the presence of suberized lenticels in apple fruit (Velickovic et al., 2014). Therefore, cuticle architecture displays chemical heterogeneities in all the dimensions of the 3D space. This architecture seems also finely regulated and mutations affecting a specific component of the cuticles induce modification of the expression of genes involves in the synthesis and assembly of the others. For example, in tomatoes, downregulation of GPAT6, an enzyme involved in the biosynthesis of the cutin substrate of *cus1*, modifies the expression of genes involved in the synthesis of polysaccharides and phenolics (Petit et al., 2016). It is also interesting to note that major cuticle-associated transcription factors, e.g., SHINE or MIXTA regulate coordinately the synthesis of polysaccharides, cutin monomer, wax and phenolics, and epidermal patterning (Shi et al., 2011; Lashbrooke et al., 2016). This fine regulation comes along with a dynamic of the cuticle architecture needed for and occurring during organ growth. This is well illustrated with the recent observation of a cutin: cutin transacylase activity capable of rearranging the architecture of the cutin polyester by a cut and paste mechanism during the growth of plant epidermis (Xin and Fry, 2021), similar to the xyloglucan endotransglucosylase/hydrolase (XTH) for polysaccharides remodeling during organ growth (Rose et al., 2002) or to the suberin polymerization-degradation process catalyzed by clusters of enzymes from the GDSL-family of esterase/lipase during lateral root formation (Ursache et al., 2021).

## From Cuticle Architecture to Functional Properties

The complexity of the architecture of plant cuticles can explain controversies in the role of the different components in their properties. This is well illustrated by the role of waxes and cutin on the water permeability of cuticles. If a consensus exists on the major role of waxes on water permeability, this is still debated for cutin. Previous studies showed differences in *Clivia miniata* leaf cuticle permeability between young and old leaves and were related to structural differences of the cutin matrix reticulation (Schmidt et al., 1981). It was suggested that wax filling of the cutin matrix depends on the cutin scaffold to explain the impact of cutin defects on cuticle water permeability (Goodwin and Jenks, 2005). However, water permeance of tomato cutin from *gpat6* tomato mutant (not affected in cutin polymerization) and from *cus1* mutants (affected in cutin polymerization) was not significantly different (Philippe et al., 2016), suggesting that the rule developed by Goodwin and Jenks cannot be applied to the cuticle of tomato fruit. Cuticle water permeability seems also related to the presence of small polysaccharide transcuticular channels, as illustrated in remnants of trichomes in some tomato accessions (Fich et al., 2020; Slot et al., 2021). The data on cuticle water permeability clearly illustrate that the functional properties of cuticles are due to the combination of the properties of each cuticular component and their hierarchical organization in a complex architecture.

Likewise, the cuticle mechanical properties are driven by the association of the cuticular components, while the direct contribution of each cuticle component is difficult to determine *in planta*. Many studies have examined the mechanical properties of isolated cuticles from different botanical origins, primarily through tensile tests (Khanal and Knoche, 2017). The resulting data demonstrated that the mechanical properties of cuticles are mainly determined by their: (i) anatomy (Allende et al., 2004; Matas et al., 2004), (ii) relative humidity (Matas et al., 2005), and (iii) relative proportions of constituents in particular during the fruit development. The accumulation of waxes and non-esterified flavonoids has been associated with an increase in cuticle stiffness (Bargel and Neinhuis, 2004; Espana et al., 2014). Notably, the contribution of esterified phenolic acids to cuticle mechanical properties is not documented although the absence of this link impairs the mechanical properties of the cuticle (Lashbrooke et al., 2016).

The cutin polyester fraction is described as a typical viscoelastic material (Knoche and Lang, 2017) whose mechanical properties would be impacted by its reticulation index. Indeed, AFM surface analyses of cutin from *cus1* tomato mutant showed a lower Young’s elastic modulus than the corresponding wild-type (Isaacson et al., 2009). This result was recently strengthened by studies on synthetic biomimetic copolyesters of cutin HOFA and glycerol where a decrease in the cutin-like polyester reticulation has been associated with a decrease in Young’s elastic modulus and a twofold increase in the strain at break (Marc et al., 2021).

The impact of different CEP on mechanical properties is less documented (Khanal and Knoche, 2017). However, paralleling the data available from the cell wall polysaccharides models, the recent identification of the specific feature of the CEP should bring new hypotheses on their impact on the cuticle mechanical properties. Indeed, reversible interactions of methyl esterified pectin with cellulose have been observed *in vitro* (Lin et al., 2016, 2018). Moreover, in pectin-cellulose model composites, an increase in the methyl esterification rate was associated with an increase in their elastic storage modulus (Lin et al., 2016, 2018). In similar model composites, hemicellulose (xyloglucan and glucomannan) affects the cellulose structuring and the mechanical properties of the composites. In particular, xyloglucan increases the composite extensibility and the decrease of its tensile elastic modulus while glucomannan leads to the opposite effect (Chanliaud et al., 2002; Berglund et al., 2020). These data open the way to the conception of new biomimetic models combining pectin, hemicellulose, and cellulose with lipids and to target specific genes in plant mutants to affect the construction and assembly of these polysaccharides and lipids to assess their specific function on the viscoelastic mechanical and/or barrier properties of cuticle.

## Conclusion

The cuticle can be considered as a polymeric composite displaying spatial heterogeneity. Our knowledge of the architecture of cuticles is rapidly progressing thanks to the development of physical instrumentation and in the future, probably with the development of correlative investigations coupling different physical techniques and modeling. Most of the studies are performed with tomato fruit which is amenable to delineate cuticle architecture. Indeed, tomato cuticle can be isolated easily, has a thickness compatible with the resolution of most physical techniques, its cutin is dominated by diOHC16 and different genetic tools are available to modify their composition. The architecture–function relationships of cuticles are still in their infancy, but should also progress rapidly and should benefit in particular (i) from the delineation of the architecture-associated enzyme network (CUS1, CXT, CCT, etc.) and (ii) from biomimicry approaches. Biomimicry will especially extend the concept of a spatially tunable architecture of the cuticle to fulfill their multiple functionalities while tailoring original bioinspired materials (Heredia-Guerrero et al., 2017).

## Author Contributions

BB, DM, and NR wrote the first draft. All authors contributed to the article and approved the submitted version.

## Conflict of Interest

The authors declare that the research was conducted in the absence of any commercial or financial relationships that could be construed as a potential conflict of interest.

## Publisher’s Note

All claims expressed in this article are solely those of the authors and do not necessarily represent those of their affiliated organizations, or those of the publisher, the editors and the reviewers. Any product that may be evaluated in this article, or claim that may be made by its manufacturer, is not guaranteed or endorsed by the publisher.
